# Dynamic monitoring of service members to quantify blast exposure levels during combat training using BlackBox Biometrics Blast Gauges: explosive breaching, shoulder-fired weapons, artillery, mortars, and 0.50 caliber guns

**DOI:** 10.3389/fneur.2023.1175671

**Published:** 2023-05-25

**Authors:** Suthee Wiri, Todd Massow, James Reid, Joshua Whitty, Cyrus Dunbar, Wallace Graves, Andrea Gonzales, David Ortley, Jasmyne Longwell, Charles E. Needham, Alex Ziegle, Virginia Phan, Fabio Leonessa, Josh L. Duckworth

**Affiliations:** ^1^Applied Research Associates, Albuquerque, NM, United States; ^2^Neurology Department, Uniformed Services University of the Health Sciences, Bethesda, MD, United States; ^3^Henry M. Jackson Foundation for the Advancement of Military Medicine, Inc., Bethesda, MD, United States; ^4^Needham Consulting, Albuquerque, NM, United States

**Keywords:** blast overpressure, operational monitoring, military training, blast sensor, body mounted

## Abstract

CONQUER is a pilot blast monitoring program that monitors, quantifies, and reports to military units the training-related blast overpressure exposure of their service members. Overpressure exposure data are collected using the BlackBox Biometrics (B3) Blast Gauge System (BGS, generation 7) sensors mounted on the body during training. To date, the CONQUER program has recorded 450,000 gauge triggers on monitored service members. The subset of data presented here has been collected from 202 service members undergoing training with explosive breaching charges, shoulder-fired weapons, artillery, mortars, and 0.50 caliber guns. Over 12,000 waveforms were recorded by the sensors worn by these subjects. A maximum peak overpressure of 90.3 kPa (13.1 psi) was recorded during shoulder-fired weapon training. The largest overpressure impulse (a measure of blast energy) was 82.0 kPa-ms (11.9 psi-ms) and it was recorded during explosive breaching with a large wall charge. Operators of 0.50 caliber machine guns have the lowest peak overpressure impulse (as low as 0.62 kPa-ms or 0.09 psi-ms) of the blast sources considered. The data provides information on the accumulation of blast overpressure on service members over an extended period of time. The cumulative peak overpressure, peak overpressure impulse, or timing between exposures is all available in the exposure data.

## 1. Introduction

Today, one of the areas of highest concern, driving research on the health impact of blast exposure, is the relationship between repeated subconcussive blast exposure and adverse neurological outcomes (1–4). In order to quantify blast exposure levels during training and combat, small, wireless blast overpressure sensors are used by researchers and environmental monitoring personnel to collect blast exposure data on military personnel during combat training (5–8). Understanding the blast environment and variability in exposure is an important step to allow researchers to correlate blast exposure (9) with physiologic changes, health impairment, and combat readiness. One such sensor is part of the Blackbox Biometrics (B3) Blast Gauge System (BGS). The BGS sensors (Blast Gauges) are designed to be worn on the body of personnel and are typically mounted on the chest, shoulder, and back of the helmet. The BGS collects overpressure vs. time data for multiple blast events which could be encountered during training events. The data available from a single blast exposure waveform include peak overpressure, peak overpressure impulse, positive phase duration, number of peaks, and timing between peaks. During training, service members are often exposed to multiple blasts and exposures are recorded by the blast sensors. The number of exposures and timing between exposures are likely factors to be considered when investigating the relationship between blast exposure and health effects. When the gauges are body-mounted, the dynamic monitoring data gives information about the blast exposure history of a service member. Service members involved in the training include students learning to use a weapon system and instructors training the students. Instructors are often exposed to multiple blasts as students rotate through the firing line during training. A research study on the effects on the brain after firing shoulder-fired weapons is looking at both trainees and instructors (10). Body-mounted blast sensors quantify the blast overpressure exposure on the surface of a subject. In general, the surface pressure values recorded by body-mounted sensors differ from the incident (free-field) blast overpressure. The difference is caused by the shock wave interaction (reflection, shielding, diffraction, etc.) with the body. The incident overpressure is the overpressure of the shock wave prior to striking an object. The reflected overpressure results from the reflection of a shock wave with a non-responding surface. The magnitude of the reflected overpressure is a function of the magnitude of the incident overpressure and the angle of incidence (11). Many blast injury thresholds are based on incident overpressure. As a result, raw body-mounted blast sensor data cannot be directly related to blast-injury correlations such as have been defined for lung (12) and ear drum rupture (13).

To address the disconnect between raw body-mounted blast sensor output and the incident overpressure parameter used in injury correlations, Wiri et al. (14) created software to estimate incident blast metrics (incident peak overpressure and incident peak overpressure impulse) using body-mounted blast sensor data as input. The Blast Gauges can also be mounted on fixed stakes to collect “static” monitoring data that can be used to better understand the blast environment around a weapon system and account for factors such as round type or position of personnel. Ultimately, both the dynamic monitoring and static monitoring data are used to better understand the blast overpressure environment around weapons and, thus, help reduce the risk of physiologic response and negative health impact by informing safer practices during training.

CONQUER (Combat and Training Queryable Exposure/Event Repository) is an operational monitoring program whose main objective is to provide reports to units and commands on the magnitude and frequency of blast exposure during training. CONQUER monitors the overpressure exposure of service members through the use of BlackBox Biometrics (B3) body-mounted sensors (Blast Gauges). CONQUER staff (Operation Managers) brief the service members, distribute the sensors, and download data from them at the end of a period of training. The data is then processed, and unit level reports are generated to provide leadership with feedback on blast overpressure exposure of members of their unit. Subject-level reports are also occasionally generated to provide more detail on the exposure of a service member. Consolidated reports, covering and comparing the blast overpressure exposure of service members across multiple units are also created for higher level military commands.

The reports increase awareness of blast overpressure exposure to commanders and can be used for comparative purposes to evaluate and quantify the impact of changes in training on the level of blast overpressure exposure. In addition, longitudinal data collected in the course of CONQUER's activity can be used to identify patterns and trends across service members from multiple services. To date, CONQUER has recorded over 450,000 gauge triggers on ~8,000 gauge sets issued to service members.

This article presents blast overpressure data recorded on B3 sensors worn by 202 Service Members during training with breaching charges, shoulder-fired weapons, artillery, mortars, and 0.50 caliber guns. These data could help direct mitigation efforts aimed to mitigate blast overpressure exposure and potentially related health risks.

## 2. Methods

The data presented in the article are a small subset of the data collected by the CONQUER program. Following the briefing of the units on the blast monitoring program, CONQUER Operations Managers distributed sensors (the B3 generation 7 BGS) to 202 service members, including both students and instructors, for dynamic monitoring of their blast overpressure exposure during training. Operations Managers trained personnel on how to use the gauges and how to place them on the back of the head, chest, and non-firing shoulder. The gauges have an adjustable trigger threshold and record a 20 ms overpressure vs. time history (waveform). For the data presented, the overpressure threshold trigger was set at values between ~3.4 and ~13.8 kPa (0.5 and 2.0 psi).

Data presented in this article are from training events with explosive breaching charges (wall, door, and window), artillery (M777 155 mm howitzer), shoulder-fired weapons (M3 MAAWS Carl Gustaf, M136 AT4 and AT4-CS, M72 LAW, and Mk 153 SMAW), mortars (M120 120 mm, M252 81 mm, and M224 60 mm), and 0.50 caliber guns (MK 15, M107, GAU-21, and M2A1). The blast sources are summarized in Table 1. The data presented are from gauges mounted on the head, chest, and shoulder. Personnel monitored under the CONQUER program might use only one weapon system. However, more commonly, a mix of weapon systems is used during a training event.

**Table 1:** Blast overpressure sources on personnel during training presented in the article.

**Explosive breaching**	**Shoulder-fired weapons**	**Artillery**	**Mortar**	**0.50 caliber gun**
Wall charge	M3 MAAWS (Carl Gustaf)	M777 155 mm	M224 60 mm	MK 15
Door charge	M136 AT4		M252 81 mm	M107
Window charge	M136 AT4-CS		M120 120 mm	M2A1
	M72 LAW			GAU-21
	Mk 153 SMAW			

The data presented were from full pressure-time waveforms (20 ms duration), and the data were reviewed to remove false positives (gauge triggers not from blast events). The peak overpressure of a waveform as well as the peak overpressure impulse of a waveform are plotted. The peak overpressure impulse is calculated by first integrating the overpressure vs. time history to obtain an overpressure impulse vs. time curve. The maximum value of the impulse curve is the peak overpressure impulse.

Scatter plots of peak overpressure vs. peak overpressure impulse were created with data and divided by blast source. Many blast sources have multiple peaks and the largest peak overpressure in a waveform is selected. Similarly, the maximum of the impulse curve is reported as the peak overpressure impulse.

A wide range of round types, propelling charge masses, and barrel angles are represented in the data. All of the data was collected during normal training operations.

## 3. Results

Data presented here represent a summary of body-mounted blast overpressure sensor data from 202 subjects, including, overall, 12,802 pressure-time waveforms. Peak overpressures range from 90.3 kPa (13.1 psi) to the minimum gauge threshold setting of 3.4 kPa (0.5 psi). The peak overpressure impulse (a measure of energy) ranges from < 0.7 kPa-ms (0.1 psi-ms; for a 0.50 caliber machine gun) to 82.0 kPa-ms (11.9 psi-ms; for a wall breaching charge).

**Figure 1:**
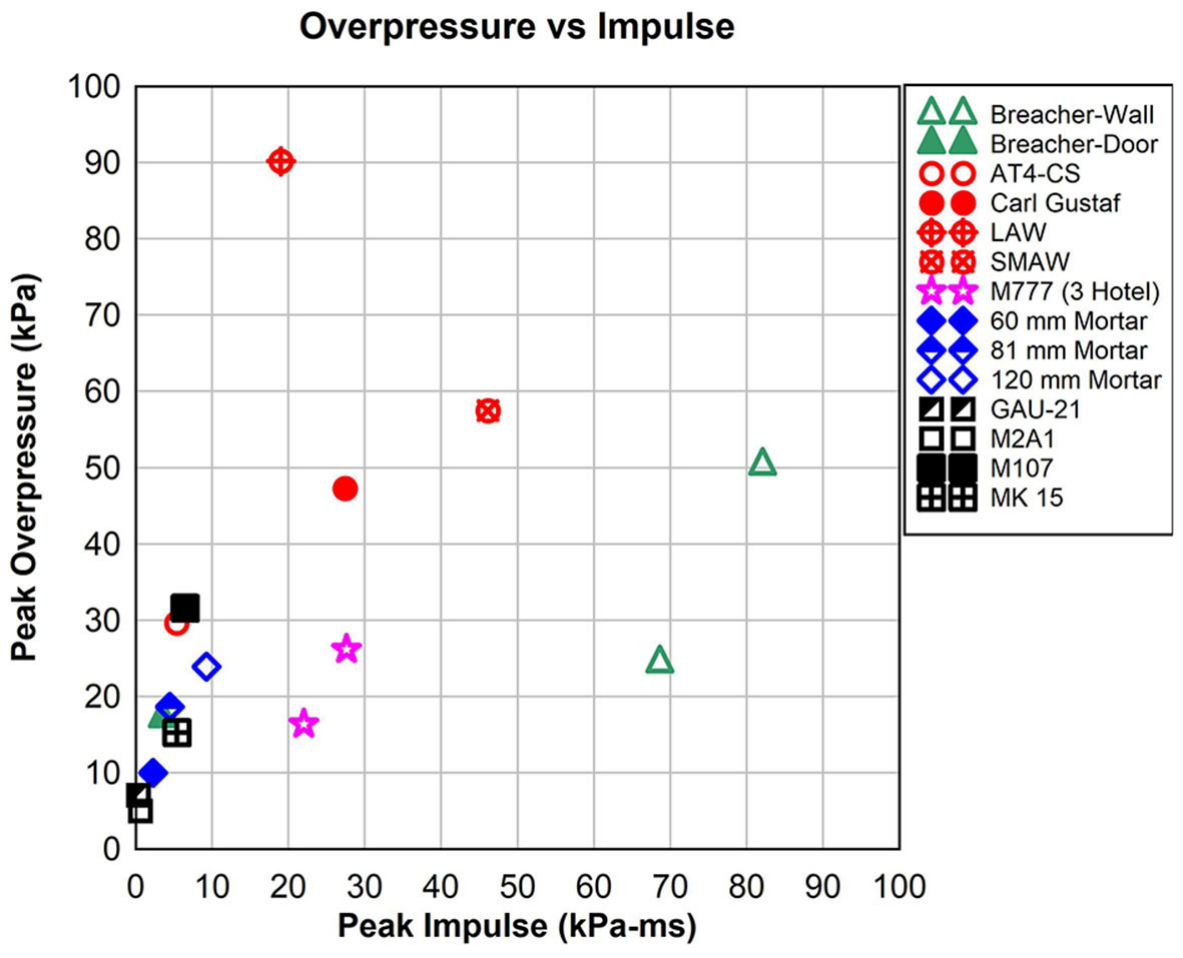
Blast overpressure and impulse (PI plot) for explosive breaching, shoulder-fired weapons, artillery, mortars, and 0.50 caliber guns. Peak overpressure and peak overpressure impulse plots show over a factor 10 range in peak overpressure and over 100 times range in peak overpressure impulse for the blast sources.

To more clearly illustrate the differences in peak overpressure (P) and impulse (I) for the blast sources, Figure 1 shows a small subset of the data. When plotted in pressure vs. impulse (PI) space, data from some of the blast sources group together. The wall charges have the largest impulse (open triangles) of 68.6 kPa-ms (9.9 psi-ms) to 82.0 kPa-ms (~12 psi-ms). Gunner and assistant gunner for shoulder-fired weapons (circles) have higher peak overpressures up to 90 kPa (~13 psi) and peak overpressure impulses between 5.5 kPa-ms (0.8 psi-ms) and 46.1 kPa-ms (6.7 psi-ms). The 0.50 caliber weapons have impulses below 6.9 kPa-ms (1.0 psi-ms). Note that the impulses for the machine guns (GAU-21 and M2A1) both have impulses below 0.7 kPa-ms (0.1 psi-ms). The impulse of the mortars correlates with the size of the barrel. The 120 mm mortar data shown has a larger impulse than the 81 or 60 mm mortars (diamonds). The 155 mm M777 artillery has peak overpressure between 16 kPa (~2.3 psi) and 27 kPa (~4 psi; stars) with impulses between 21 kPa-ms (~3 psi-ms) and 28 kPa-ms (~4.1 psi-ms).

**Figure 2:**
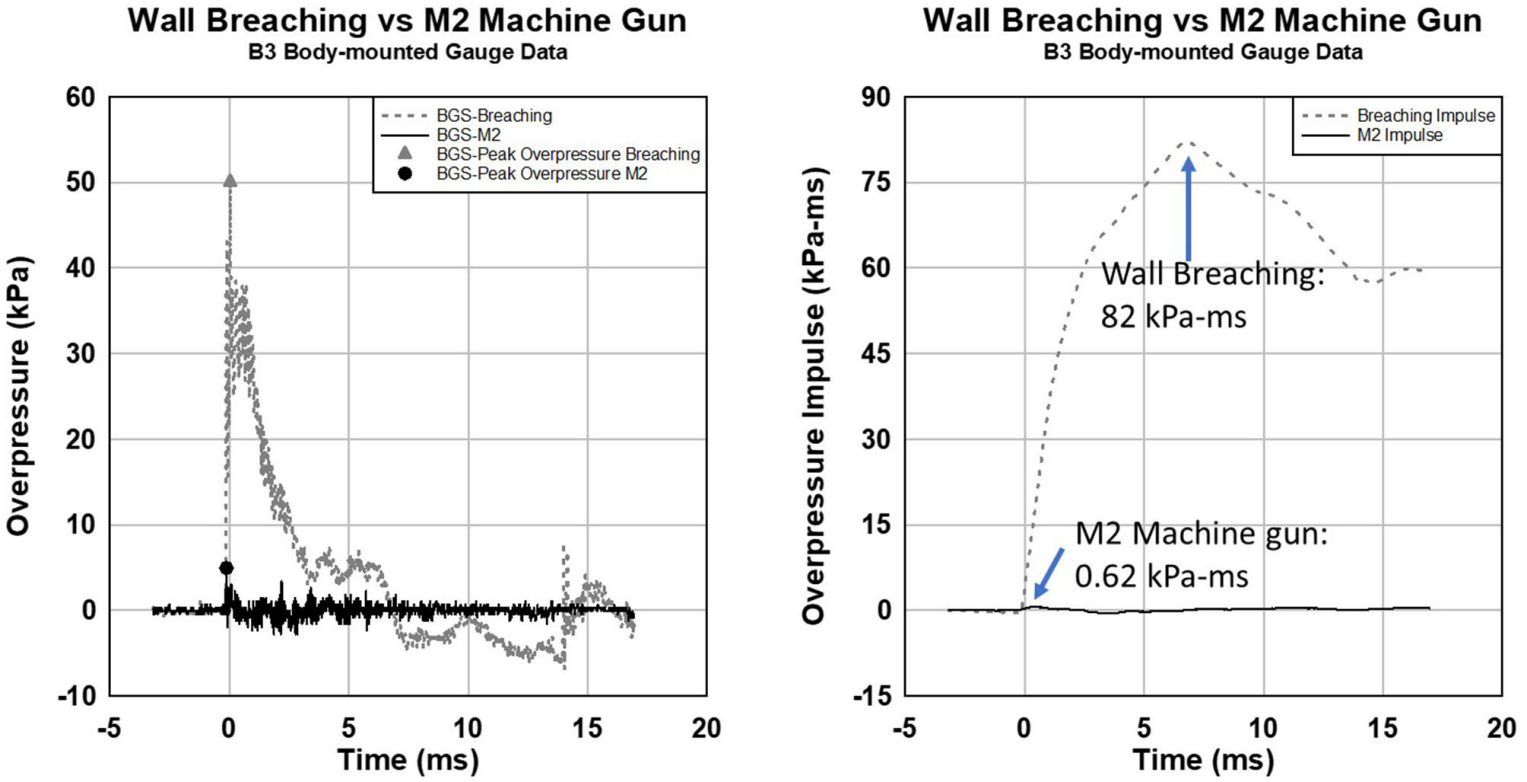
Comparison of blast overpressure **(left)** and impulse **(right)** for explosive wall breaching and M2A1 machine gun. Peak overpressure for wall breacher and M2A1 is 50.3 and 4.8 kPa, respectively. Peak overpressure impulse is 82.0 and 0.62 kPa-ms (factor of 130) for breacher and M2A1.

The data shown in Figure 1 has peak overpressures that differ by over a factor of 10 and peak overpressure impulse varies by over 100 times. Each blast exposure has an overpressure vs. time history and select values are used to illustrate the characteristic blast exposure levels. Two waveforms representing the largest and smallest impulse are shown in Figure 2. The wall breaching charge has a peak overpressure of 50.3 kPa (7.3 psi) while the M2A1 machine gun peak overpressure was 4.8 kPa (0.7 psi). The impulse of the wall charge (82.0 kPa-ms or 11.9 psi-ms) is 130 times larger than the M2 machine gun (0.62 kPa-ms or 0.09 psi-ms) as shown on the right.

**Figure 3:**
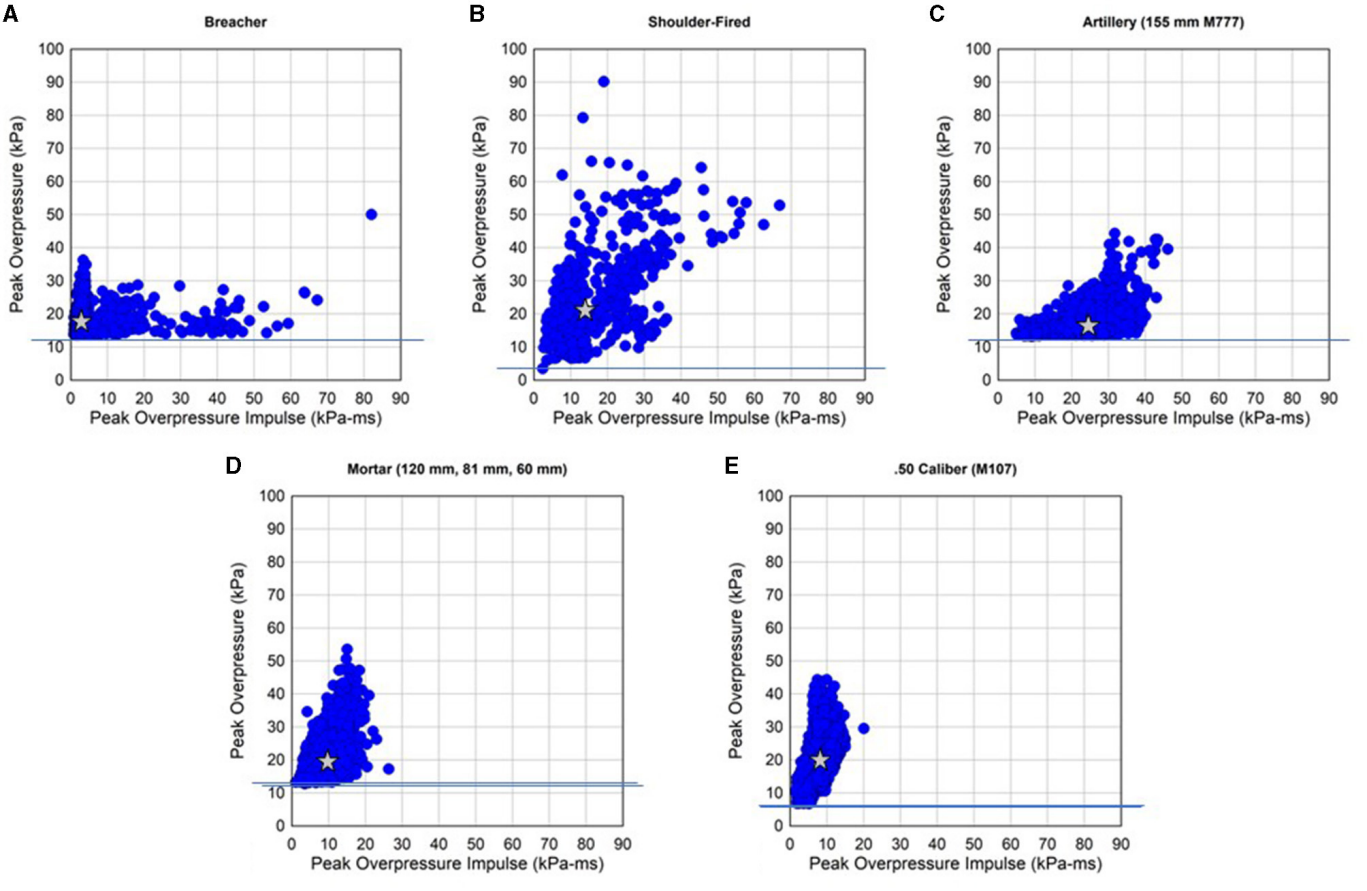
Body-mounted blast sensor data peak overpressure and peak overpressure impulse from **(A)** explosive breaching, **(B)** shoulder-fired weapons (M3 Carl Gustaf, AT4 & AT4-CS, LAW, and SMAW), **(C)** artillery (M777), **(D)** mortars (120, 81, and 60 mm), and **(E)** 0.50 sniper (M107) training.

Scatter plots of peak overpressure and peak overpressure impulse for all 202 subjects and 12,802 waveforms for the five blast sources are shown in Figure 3. The variability in the data is due in part to the different weapon systems, charge type, round type, subject position, and subject body orientation. The data was collected during normal training exercises. The breakdown of subjects and the number of waveforms for the explosive breaching, shoulder-fired weapons, artillery, mortars, and 0.50 caliber guns are shown in Table 2. The table includes data from head, chest, and shoulder gauges. The waveform with the largest peak overpressure was selected and the value is given. Similarly, the waveform with the largest peak overpressure impulse was selected and the values are shown in Table 2. The 0.50 cal sniper rifle has the highest average number of waveforms per subject at 131.3. The average number of waveforms per subject for shoulder-fired weapons was 8.2.

**Table 2:** The number of subjects, waveforms, median peak overpressure, median peak overpressure impulse, maximum peak overpressure, and maximum peak overpressure impulse from the body-mounted blast overpressure sensor data.

**Training**	**Number of subjects**	**Number of waveforms**	**Average Number of waveforms per subject**	**Median number of waveforms per subject**	**Median peak over-pressure (kPa)**	**Median peak over-pressure impulse (kPa-ms)**	**Maximum peak over-pressure (kPa)**	**Maximum peak over-pressure impulse (kPa-ms)**	**Average number of days between first and last exposure**	**Average number of days with exposures**
Explosive breaching	29	2,095	72.2	56	18.0	2.7	50.3	82.0	240	16
Shoulder-fired weapons (Carl Gustaf, AT4 & AT4-CS, LAW, and SMAW)	55	452	8.2	6	21.5	13.7	90.3	66.9	13	1
Artillery (155 mm M777)	57	2,703	47.4	32	16.6	24.3	44.1	46.2	66	7
Mortars (120, 81, and 60 mm)	25	2,826	113.0	75	19.7	9.4	53.8	26.2	112	2
0.50 sniper rifle (M107)	36	4,726	131.3	137.5	20.1	7.8	44.1	20.0	41	2

The duration of the training and the number of days with blast exposures differ between the weapon systems. The average duration (number of days) between the first and last blast gauge trigger for the subjects is shown in Table 2. Then the average number of days with a blast gauge trigger is shown for each training type. For example, the shoulder-fired weapon average was 13 days between the first and last gauge trigger, and the average number of days with a blast gauge trigger was 1 (indicating that the training usually occurred in 1 day). However, explosive breaching had 240 days between the first and last trigger and an average of 16 days with gauge triggers.

The explosive breaching scatter plot shows the highest impulse at 82.0 kPa-ms (11.9 psi-ms; Figure 3A). The majority of the data has a peak overpressure of < ~28 kPa (4.0 psi). However, when the impulse is < 6.9 kPa-ms (1.0 psi-ms) there are gauge triggers with peak overpressures above 34 kPa (~5 psi). The shoulder-fired weapon data has the highest peak overpressures with many triggers above 41 kPa (~6 psi; Figure 3B). The impulses are also high with many readings above 62 kPa-ms (~9 psi-ms). The M777 155 mm artillery data is mainly clustered between 21 kPa-ms (~3 psi-ms) and 41 kPa-ms (~6 psi-ms), and the vast majority of the data has peak overpressure < ~28 kPa (4.0 psi). The mortar systems have impulses that are less than about 21 kPa-ms (~3 psi-ms). However, a large number of peak overpressures exceeding ~28 kPa (4.0 psi) are present with some blast exposure magnitudes up to 53.6 kPa (7.8 psi). The M107 sniper training had the lowest impulses (blast energy) with the vast majority of the data below 15 kPa-ms (~2.2 psi-ms). The peak overpressures of the M107 did have values exceeding 41 kPa (~6 psi). The data in Figure 3 is taken from the head, chest, and shoulder gauges. The stars in the plots correspond to the median peak overpressure and median peak overpressure impulse for each of the blast sources. The shoulder-fired weapon, 0.50 cal sniper, and mortars all have a median peak overpressure of around 20 kPa (ranging from 19.7 to 21.5 kPa). The artillery has the highest peak overpressure impulse (24.3 kPa-ms), but the lowest median peak overpressure (16.6 kPa). Explosive breaching has the lowest median peak overpressure impulse (2.7 kPa-ms). The median values are listed in Table 2.

The minimum peak overpressure recorded varies due to differences in the threshold used on the BGS. The threshold was ~13.8 kPa (2.0 psi) for breaching, M777, and mortars, but was set at ~3.4 kPa (0.5 psi) for M107 and shoulder-fired weapons (as denoted by the horizontal lines on the figures).

In the following sections, body-mounted blast overpressure sensor data are presented for multiple blast sources from combat training. The waveforms show the full overpressure vs. time history recorded by the gauges. In many cases, there are multiple peaks in the waveform.

### 3.1. Explosive breaching

A wall explosive breaching charge can have a net explosive weight (NEW) of ~7.8 kg (17 lb.). Door charges can have a 90 g (0.2 lb.) NEW. The range in explosive weight and standoff distance explain much of the variation in peak overpressure and impulse shown in Figure 3A. The data was collected in an unsupervised mode, and Operation Managers were not monitoring and recording details of the blast events such as standoff distance, charge size, or subject positioning.

**Figure 4:**
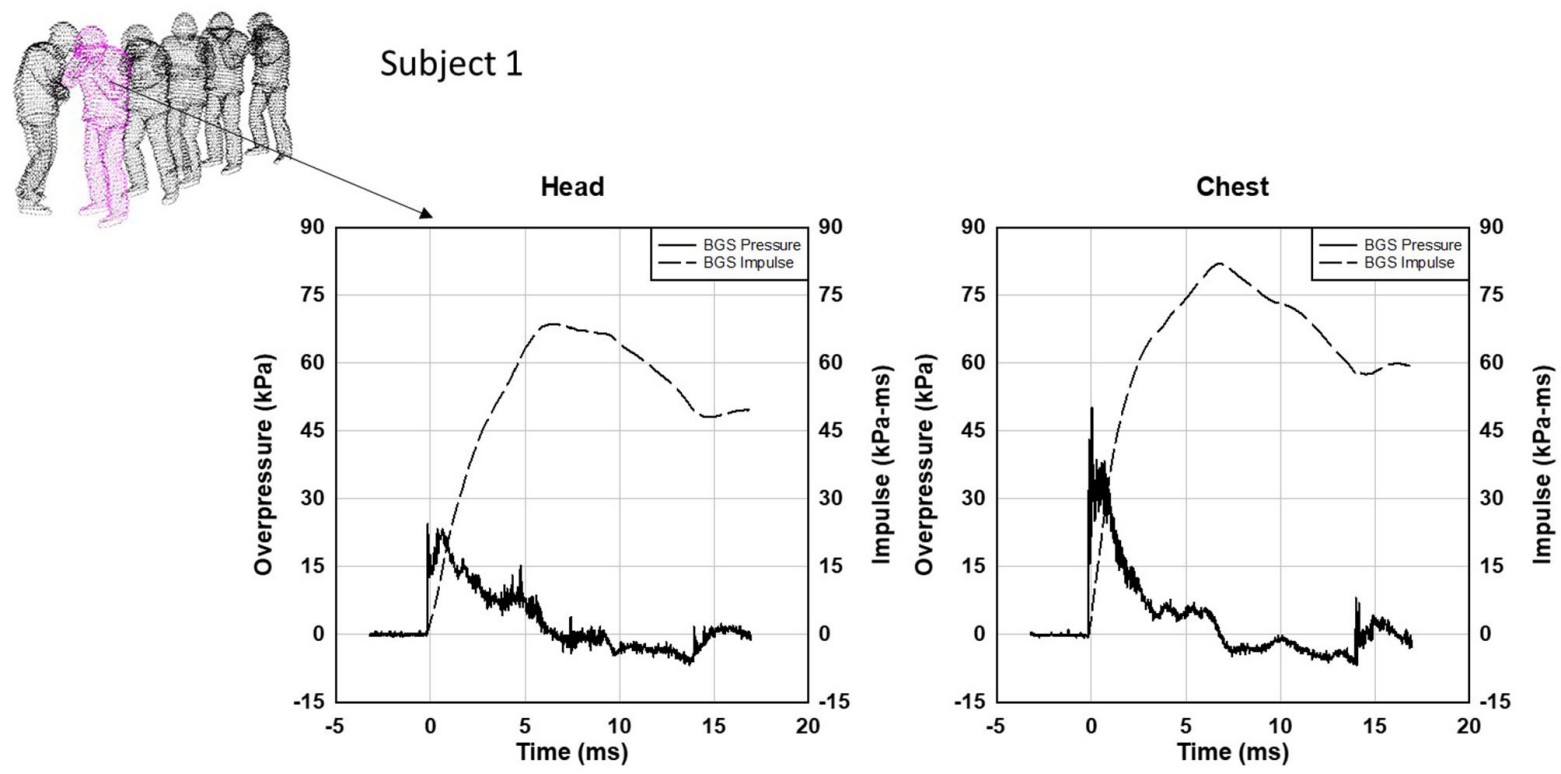
Body-mounted blast sensor data for Subject 1 from explosive wall breaching. The chest gauge records a higher peak overpressure (50.3 kPa) because of a blast reflection on the body. The head gauge mounted on the back of the head is shielded by the head, so a lower peak overpressure is recorded (24.1 kPa).

For the scenario below, CONQUER Operation Managers observed the explosive breaching event, and the data collected from a wall charge represents the largest impulse blast exposures recorded by CONQUER during supervised monitoring (Figure 4). Six subjects were stacked near a 7.8 kg (17 lb.) NEW wall breaching charge. Subject 1 was facing the blast so, due to a shock reflection, the chest gauge recorded the highest peak overpressure of the gauges at 50.3 kPa (7.3 psi). The head gauge (placed on the nape of the neck) was shielded from the blast by the head and recorded 24.1 kPa (3.5 psi). The peak overpressure impulse at the head was 68.9 kPa-ms (10.0 psi-ms) and the chest was 82.0 kPa-ms (11.9 psi-ms). The positive phase duration was over 6 ms for both gauges.

### 3.2. Shoulder-fired weapons

The waveforms associated with operating shoulder-fired weapons typically have multiple peaks. Since the subjects are holding the weapon and are very close to the source of the blast, the peak overpressure can be over 68 kPa (~10 psi). There was no large dataset with only shoulder-fired weapons so one was created using smaller training events with the M3 MAAWS (Carl Gustaf), M72 LAW, M136 AT4 & AT4-CS, and Mk153 SMAW.

**Figure 5:**
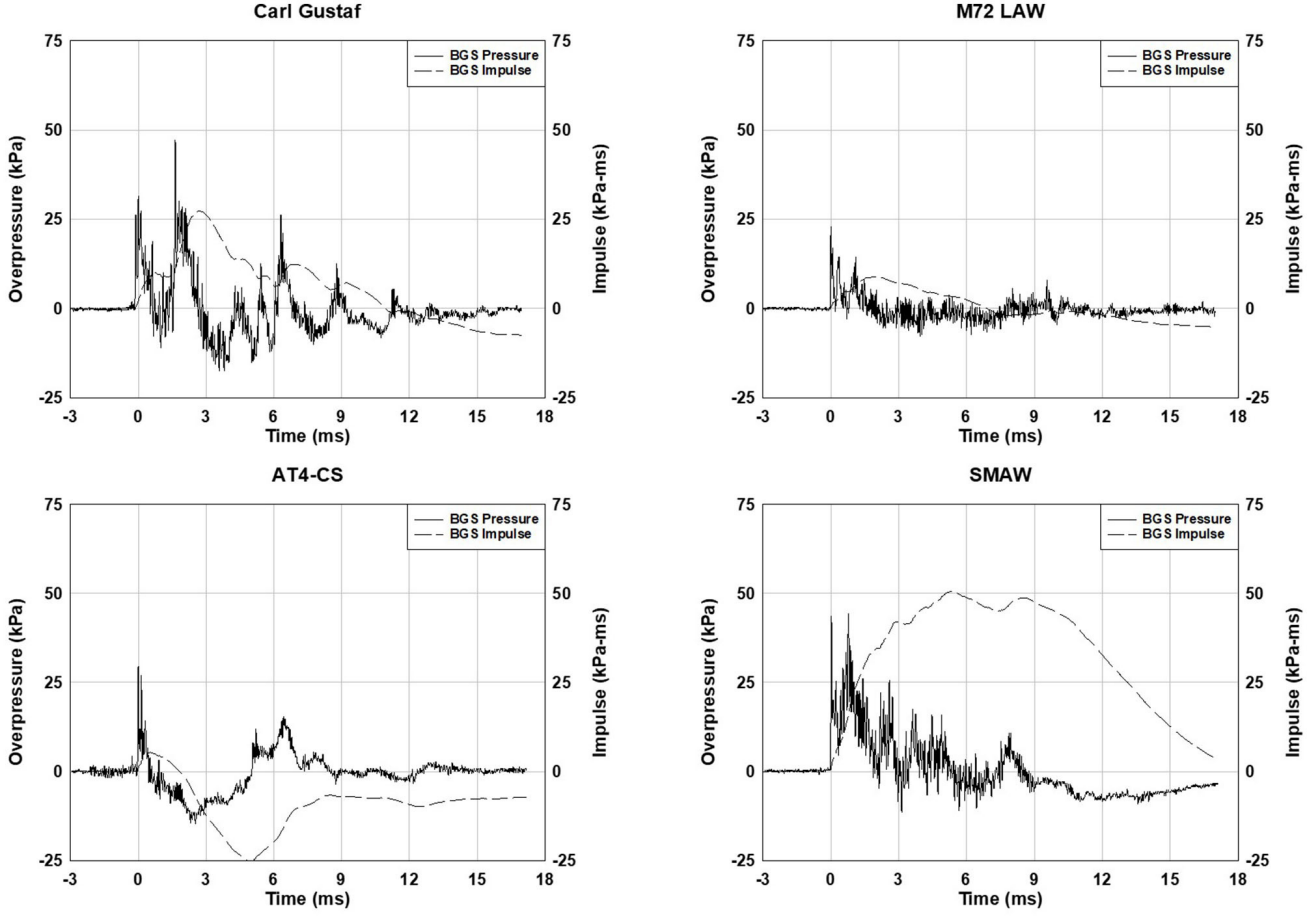
Body-mounted blast sensor data from the head of the gunner from M3 MAAWS Carl Gustaf, M72 LAW, M136 AT4-CS, and Mk 153 SMAW shoulder-fired weapons. The waveform shapes vary with weapon type.

Waveforms from head sensors for the shoulder-fired weapons are shown in Figure 5. The blast overpressure exposure is a function of round type, position relative to the weapon, and body position. The waveforms are examples of blast exposure data collected by gunners of shoulder-fired weapons. The peak overpressure impulse of the SMAW exposure shown is over 50 kPa-ms (7.3 psi-ms). The AT4-CS peak overpressure impulse is around 5.3 kPa-ms (~0.8 psi-ms).

### 3.3. Artillery

The blast exposure to operators of the 155 mm artillery M777 typically has two peaks. The first is an incident blast wave from the muzzle, and the second is a ground reflection. The timing and magnitude of the ground reflection depend on the barrel angle (quadrant and traverse) and position of the subject. The magnitude of the blast exposure increases as the propelling charge mass increases. The 5 HOTEL (M232A1) charge was the largest propelling mass where data were collected. The 2 LIMA (M232) charge shows a much lower blast exposure magnitude than a 5 HOTEL charge.

Data from 155 mm artillery (M777) training are shown in Figure 3C. The maximum peak overpressure was 44.4 kPa (6.4 psi) from the 57 subjects and 2,703 waveforms. In this case, the data collection was not monitored so the subject position, propelling charge mass, and other variables were not recorded.

**Figure 6:**
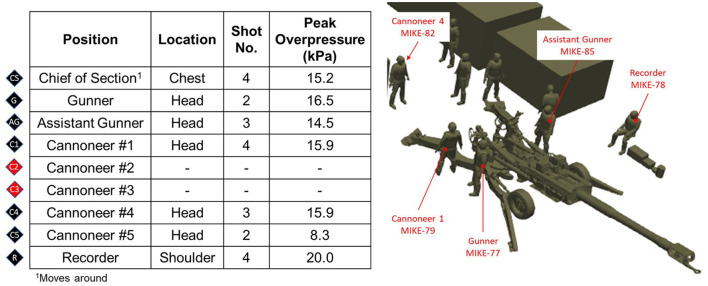
Overview of peak overpressure and peak overpressure impulse for subjects around a 155 mm howitzer (M777). The propelling mass was 3 HOTEL (M232A1).

A data collection where Operation Managers recorded the conditions is shown in Figure 6. Nine subjects were around the M777 when firing an M795 round with a 3 HOTEL (M232A1) propelling charge. The peak overpressure for the head gauge of each subject varies between 20.0 kPa (2.9 psi) and 8.3 kPa (1.2 psi). No gauge data was recorded for Cannoneer #2 and #3.

**Figure 7:**
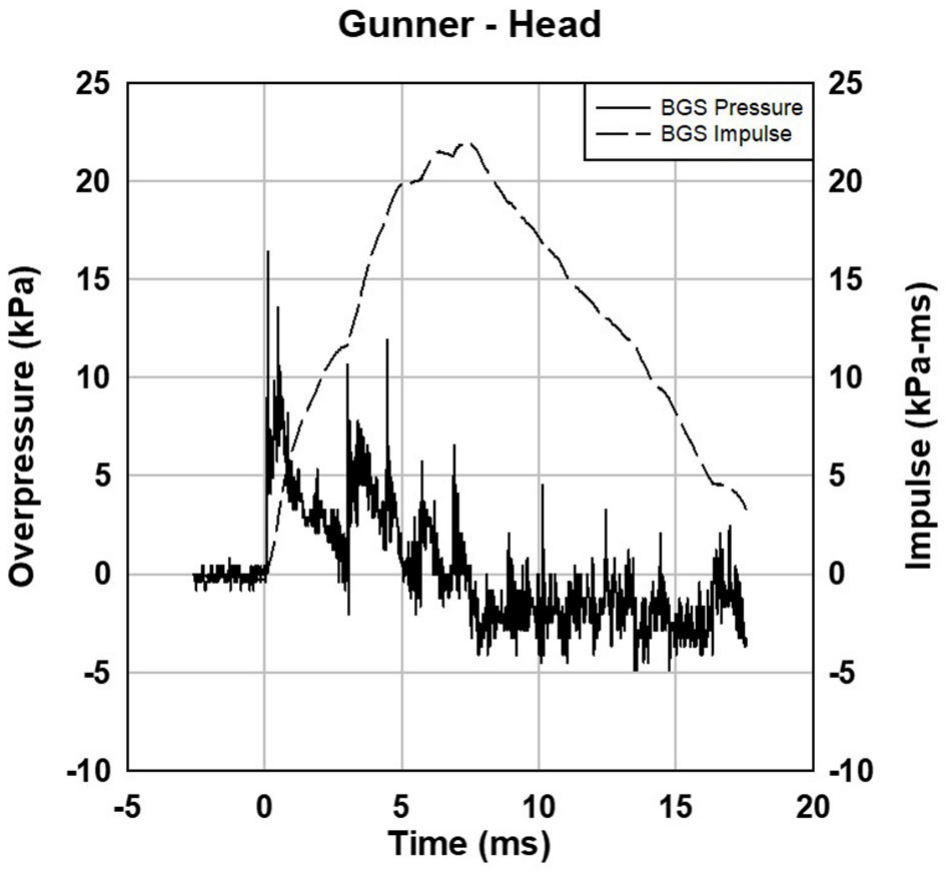
Gunner head gauge data for 3 HOTEL M777. The two peaks are from the incident blast wave and ground reflection.

Head gauge waveform data for the gunner is shown in Figure 7. There are two peaks in the waveform from the incident muzzle blast and a ground reflection.

### 3.4. Mortars

Body-mounted blast sensor data from mortar training for 25 subjects shows the variation in peak overpressure and peak overpressure impulse (Figure 3D). A total of 2,826 waveforms are shown. Recall that up to three waveforms (head, chest, and shoulder) can be recorded for each blast exposure. The mortar systems used during training include the 60, 81, and 120 mm mortars.

**Figure 8:**
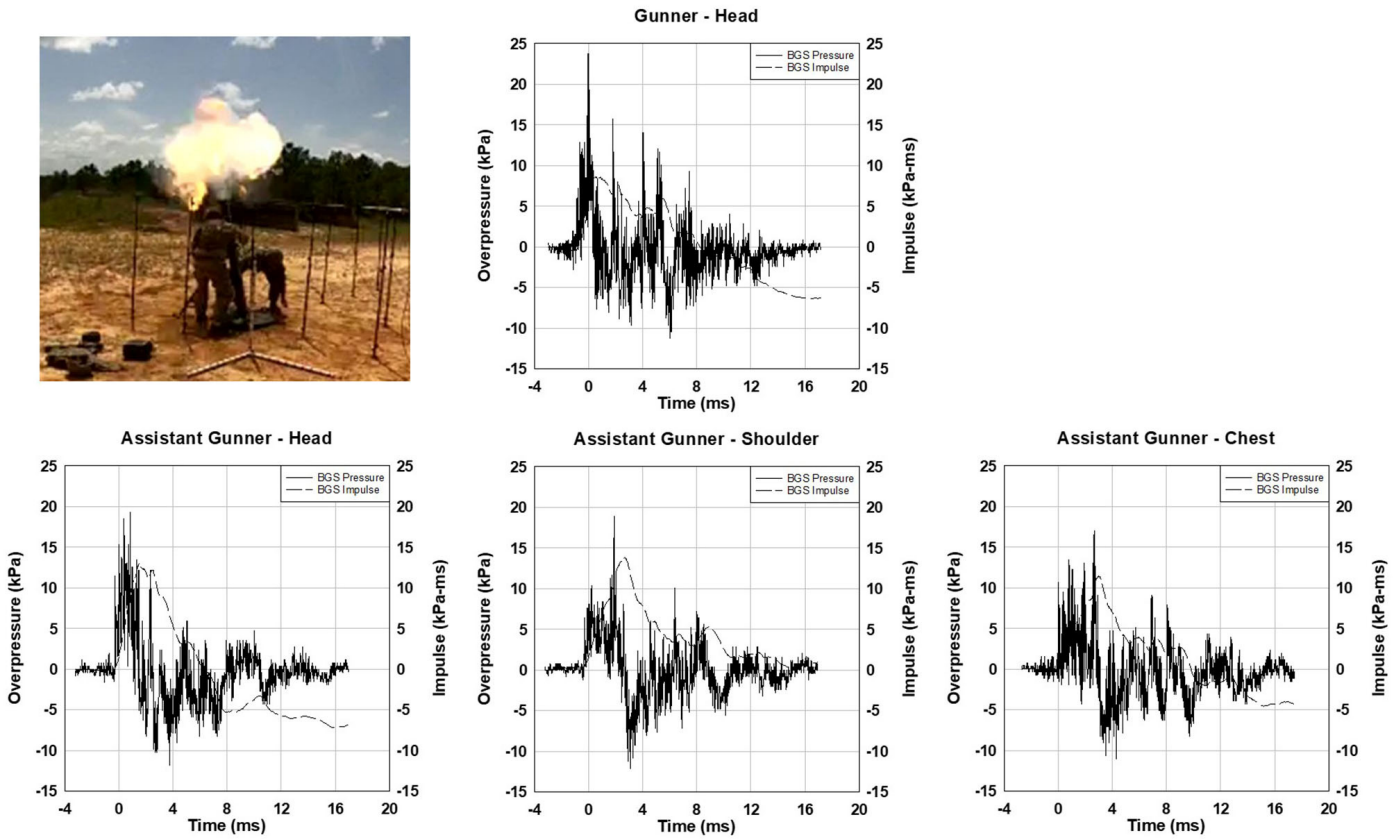
Photo of the gunner (standing) and assistant gunner (bending down) around a 120 mm mortar. The plot of overpressure vs. time histories for gunner (top) and assistant gunner (bottom) for 120 mm mortar with 1 M230 propelling charge.

A data collection where Operation Managers recorded the conditions is shown in Figure 8. Two subjects were operating a 120 mm mortar with an M933 HE mortar cartridge with 1 M230 propelling charge. The peak overpressure for the head gauge of the gunner was 23.4 kPa (3.4 psi) while the assistant gunner's head gauge recorded 19.3 kPa (2.8 psi). Note the waveforms have multiple peaks with varying positive phase duration.

### 3.5. 0.50 caliber weapons

Body-mounted blast sensor data from M107 sniper training is shown in Figure 3E. The sniper and spotter wore gauges during the training. Blast exposures came from operating the weapon or blast exposures from snipers in adjacent lanes.

Occasionally, very high peak overpressures of up to 110 kPa (~16 psi) were recorded during sniper training. However, upon further investigation, it was determined that the subject was not wearing the gauges at the time of the high magnitude exposure. Rather the body armor (kit) with Blast Gauges was placed on the bench as a rest for the rifle during firing. As a result, the blast sensors were very close to the muzzle blast and recorded high peak overpressures. This happened multiple times and illustrates that blast data recorded is not necessarily a blast exposure on the subject since in this case the gauges were not being worn by the subject.

**Figure 9:**
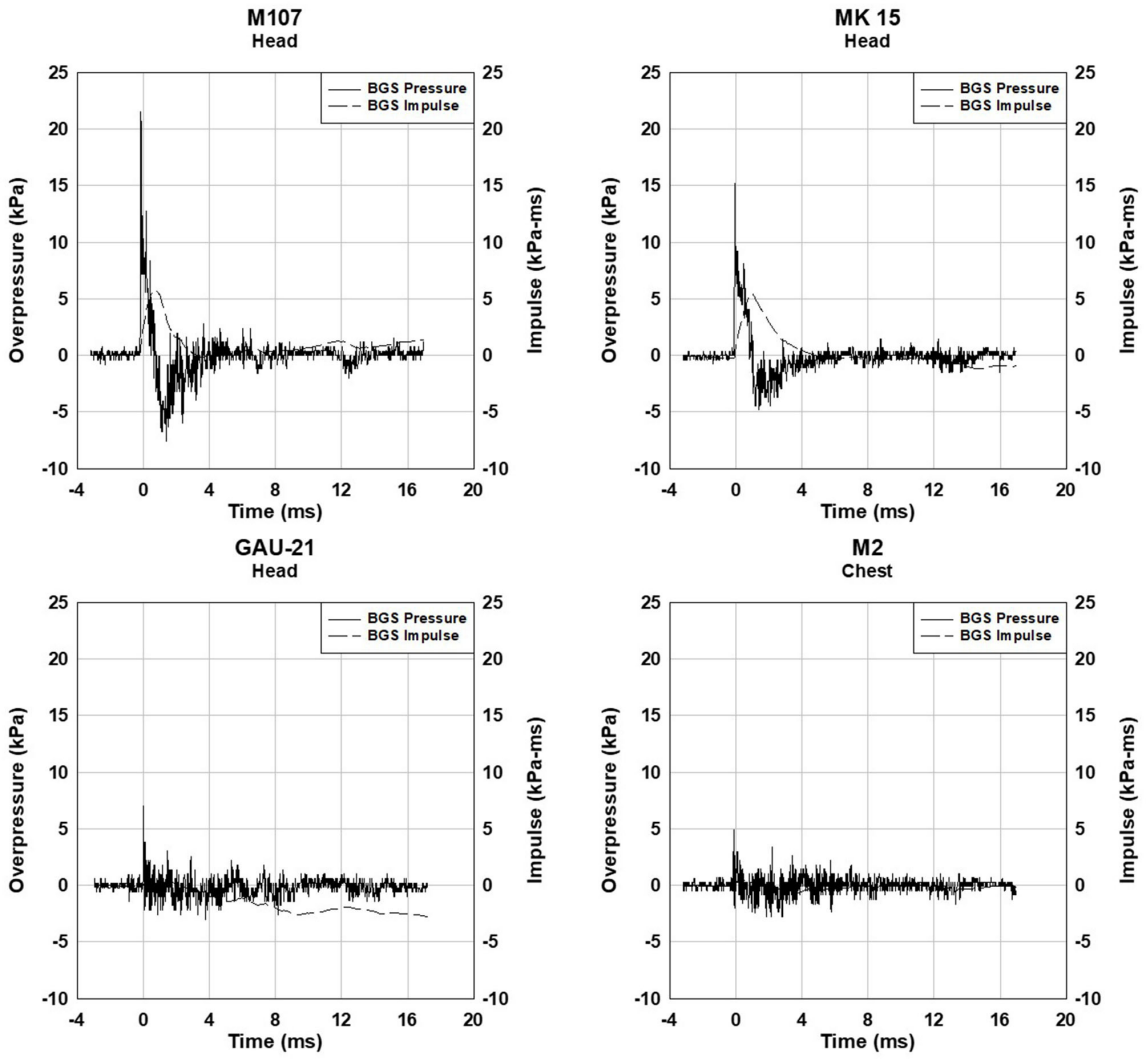
Blast overpressure data from 0.50 caliber weapons at the head gauge for the MK 15, M107, and GAU-21 and the chest gauge for the M2A1.

Data from four 0.50 caliber weapons (M107, MK 15, M2A1, and GAU-21) were collected. The barrel length, muzzle brake, or flash suppressor all influence the resulting blast exposure on the operator. Example waveforms from the four weapons are shown in Figure 9.

The blast exposures associated with 0.50 caliber weapons have lower impulse (energy) than other weapon systems such as 120 mm mortars, M777 artillery, and shoulder-fired weapons. A 60 mm mortar or small explosive breaching charge could have blast exposure levels of similar magnitude impulse to 0.50 caliber weapons. The other consideration with 0.50 caliber machine guns is the number of blast exposures could be much higher than in other weapon systems. Automatic guns could expose the operators to hundreds or even thousands of low-level repeated blast exposures.

## 4. Discussion

To date, the CONQUER program has collected over 230,000 waveforms on service members during training. Over 450,000 gauge triggers including both waveforms and summary events have been recorded. The data provide information on blast exposure profiles including magnitude and frequency to commanders and researchers. In general, the raw body-mounted blast sensor data do not represent incident overpressure typically used in blast injury correlations. Therefore, the peak overpressure and peak overpressure impulse values presented are not suitable for use as input into blast injury correlations that require incident blast as input. The software has been developed to estimate incident blast metrics using body-mounted blast sensor data (14). Both peak overpressure and peak overpressure impulse (a measure of blast energy) data are presented since both could be important for correlation with physiologic changes. All overpressure waveforms include the negative phase overpressure and impulse. The maximum negative overpressure is always less than the peak positive overpressure, while the negative impulse is nearly equal to that of the positive phase. The effects of the negative phase could be more benign because of the lower magnitude of the pressure and the onset is much more gradual compared to the rise associated with the shock.

The number of blast exposures over time (within a day, month, or year) is expected to have an effect on adverse neurologic outcomes, but the relationship between peak overpressure, peak overpressure impulse, and number of exposures is not yet known. For example, the 0.50 cal sniper rifle's median peak overpressure impulse (a measure of energy) is 7.8 kPa-ms while the shoulder-fired weapon's median peak overpressure impulse is 13.7 kPa-ms an increase of 75.6%. The timescale of repeated blast exposures spans milliseconds to years and is a variable that must be considered when developing correlations between blast overpressure and physiologic changes.

Patterns and trends in blast exposure across the units are also observed. Comparison of the blast exposures between units conducting similar training could identify approaches to mitigate blast exposure. The variances in training environments, weapon systems used, protocols, and individual (behavioral) blast exposure can be studied. The variation in blast exposure within a unit has been used to differentiate blast exposure levels between subjects and identify the variables leading to increased exposure. Once identified, measures can be implemented to reduce blast exposure in the future.

Mapping blast exposure environments in training identifies potential high overpressure regions around a weapon system. Once the regions are identified, one can mitigate blast exposure. For example, during explosive breaching training, the number of charges detonated per day could be reduced or the standoff distance could be increased. This process is especially useful for indoor ranges where there are multiple shock reflections from the walls, floor, and ceiling. The CONQUER program mapped multiple ranges and units increased standoff distance based partly on data collected by body-mounted blast sensors.

In several cases, personnel monitoring by CONQUER was successfully used to mitigate exposure. Blast overpressure exposure data reported to the units led to changes such as increased lane spacing between trainees and adjustment of range safety officers positioning during shoulder-fired weapon training (reducing the magnitude of each blast overpressure exposure). Other induced changes included a reduction in the number of rounds fired per day thus distributing exposures over more days (reducing daily cumulative blast exposure).

One limitation is that monitoring is a passive, indirect data collection process, such that, without direct surveillance, there is uncertainty in the context of the data and the application of the data is limited. Examples include gauges that are either being used in an unanticipated way such as being on kits while being used as a weapon support during sighting in or gauges either intentionally or unintentionally exposed to blast environments or other pressure or mechanical forces without association with the assigned individual. The CONQUER program addressed this by collecting training schedules and noting weapon systems used during training. Without direct observation, environmental factors such as temperature, elevation, humidity, wind, and other physiologic factors such as other exposures, noise, secondary, tertiary, blast effects, and physical and psychological stress factors are not collected.

A possible solution to passive monitoring is “smart” active monitoring that allows for a direct or better understanding of the situation under which the data was obtained or a larger known dataset from which exposure data can be compared and matched to the best fit, not just for weapon type but also a vector, position, protection/shielding, conditions, etc.

Another limitation of body-mounted blast sensors is the compliance of wearing the sensors during training. Some service members do not wear their gauges. However, if the service members are briefed about the function of the sensors and the reason for monitoring, they were more likely to wear the sensors. Another advancement to increase compliance could be integrating sensors into the service member's protective equipment. This could decrease the number of lost gauges.

Future efforts could include research studies on the service members and blast sources that are more likely to have adverse neurologic effects. A major priority for future efforts is the definition of thresholds for traumatic brain injury and adverse physiologic response to blast overpressure. Development of a dose response curve that accounts for the magnitude of blast exposure, number of peaks within a blast exposure, number of exposures, time between exposures, the effect of personal protective equipment, etc. should be the goal for the community. Advancements in correlations to physiologic outcomes could be used to model different scenarios and training evolutions to allow service members to train in a safer manner. Fast-running software tools that provide near-real time predictions of blast exposure when training could facilitate the prediction of expected overpressure exposure when training with weapons or activities like explosive breaching.

## 5. Conclusion

The data presented show the differences in peak overpressure and peak overpressure impulse (a measure of energy) from five different classes of blast sources. Data from body-mounted blast overpressure sensors were presented including over 12,000 waveforms on 202 subjects. The magnitude and frequency of blast exposure were collected for each subject in training. On average, shoulder-fired weapons have the highest peak overpressures. On average, the 0.50 caliber machine guns have the lowest impulse. The highest impulse blast exposures in the collected data are from explosive breaching wall charges. The peak overpressure range of the M107 sniper rifle and mortars are similar. However, the mortars have a higher magnitude impulse than the M107.

There can be multiple peaks in a blast overpressure waveform for operators of shoulder-fired weapons and mortars. The presence of multiple blast waves within a 20 ms waveform has been observed and validated in the data. For artillery training, blast exposures tend to have two peaks from an incident blast and a ground reflection. The 0.50 caliber guns usually have a single peak with a smaller peak overpressure impulse than the other blast sources considered.

The variability in both peak overpressure and peak overpressure impulse is shown in the data. The subject location, gauge mounting location on the body, distance from the blast source, and angle relative to the source (some sources have angle dependent blast magnitudes) all affect the blast exposure for a given blast source. The data shows the relative magnitude of peak overpressure and impulse for the five blast sources. Potential mitigation approaches are described. In several instances, the CONQUER program's blast overpressure reports to units induced the adoption of successful exposure measures including an increase in standoff distance, adjustment of body position, and increase in the time interval between firings/exposures.

## Data availability statement

The datasets analyzed during the current study are not publicly available due to controlled unclassified information but are available upon reasonable request. Requests to access these datasets should be directed to fabio.leonessa.ctr@usuhs.edu.

## Ethics statement

Ethical review and approval was not required for the study on human participants in accordance with the local legislation and institutional requirements. Written informed consent from the patients/participants or patients/participants' legal guardian/next of kin was not required to participate in this study in accordance with the national legislation and the institutional requirements.

## Author contributions

AG, DO, JL, AZ, and VP performed the data analysis. SW, FL, and JD wrote the manuscript in consultation with CN. SW, TM, JR, JW, CD, WG, FL, and JD involved in planning and performing the data collection. SW conceived of the presented idea. JD conceived the project idea and supervised the project. All authors contributed to the article and approved the submitted version.

## Author disclaimer

The opinions and assertions expressed herein are those of the author(s) and do not necessarily reflect the official policy or position of the Uniformed Services University or the Department of Defense. The contents of this publication are the sole responsibility of the author(s) and do not necessarily reflect the views, opinions, or policies of The Henry M. Jackson Foundation for the Advancement of Military Medicine, Inc. Mention of trade names, commercial products, or organizations does not imply endorsement by the U.S. Government.
